# Are There Epigenetic Oxytocin-Mediated Effects on the Mother and Infant during Physiological Childbirth?

**DOI:** 10.3390/ijms21249503

**Published:** 2020-12-14

**Authors:** Kerstin Uvnäs-Moberg, Mechthild M. Gross, Andee Agius, Soo Downe, Jean Calleja-Agius

**Affiliations:** 1 Department of Animal Environment and Health, Swedish University of Agricultural Sciences, 53223 Skara, Sweden; kerstinuvnasmoberg@gmail.com; 2 Midwifery Research and Education Unit, Hannover Medical School, 30625 Hanover, Germany; gross.mechthild@mh-hannover.de; 3 Department of Obstetrics and Gynaecology, Mater Dei Hospital, MSD2090 Msida, Malta; andeeagius@yahoo.com; 4 School of Community Health and Midwifery, University of Central Lancashire, Preston PR1 2HE, UK; SDowne@uclan.ac.uk; 5 Department of Anatomy, Faculty of Medicine and Surgery, University of Malta, MSD2080 Msida, Malta

**Keywords:** oxytocin, oxytocin receptor, epigenetics, polymorphisms, vaginal birth, caesarian section, skin-to-skin contact, effects of oxytocin, longterm effects, health

## Abstract

Introduction: Studies have shown that long-term positive behavioural and physiological changes are induced in connection with vaginal, physiological birth, and skin-to-skin contact after birth in mothers and babies. Some of these effects are consistent with the effect profile of oxytocin. This scoping review explores whether epigenetic changes of the oxytocin gene and of the oxytocin receptor gene (OTR) are involved in these effects. Methods: We searched Pubmed, Medline, BioMed Central, Cochrane Library, OVID, and Web of Science for evidence of epigenetic changes in connection with childbirth in humans, with a particular focus on the oxytocin system. Results: There were no published studies identified that were related to epigenetic changes of oxytocin and its receptor in connection with labour, birth, and skin-to-skin contact after birth in mothers and babies. However, some studies were identified that showed polymorphisms of the oxytocin receptor influenced the progress of labour. We also identified studies in which the level of global methylation was measured in vaginal birth and caesarean section, with conflicting results. Some studies identified differences in the level of methylation of single genes linked to various effects, for example, immune response, metabolism, and inflammation. In some of these cases, the level of methylation was associated with the duration of labour or mode of birth. We also identified some studies that demonstrated long-term effects of mode of birth and of skin-to-skin contact linked to changes in oxytocin function. Conclusion: There were no studies identified that showed epigenetic changes of the oxytocin system in connection with physiological birth. The lack of evidence, so far, regarding epigenetic changes did not exclude future demonstrations of such effects, as there was a definite role of oxytocin in creating long-term effects during the perinatal period. Such studies may not have been performed. Alternatively, the oxytocin linked effects might be indirectly mediated via other receptors and signalling systems. We conclude that there is a significant lack of research examining long-term changes of oxytocin function and long-term oxytocin mediated adaptive effects induced during physiological birth and skin-to-skin contact after birth in mothers and their infants.

## 1. Introduction

Animal experiments have shown that pregnancy, birth, and the early postpartum period were associated with adaptive physiological and behavioural adaptations [1,2] Stress, during these periods, could result in life long stress sensitivity [3], whereas increased levels of tactile interaction and closeness could result in long-term increase in social competence, decreased levels of anxiety and stress, as well as stimulation of processes related to restoration and growth [4,5]. Epigenetic mechanisms have been implicated in these long-term effects [6].

Studies performed in humans have indicated that, on the one hand, normal stress (eustress) induced by physiological birth could be associated with beneficial and adaptive effects in both mother and infant [7]. For example, lung maturation is stimulated by elevated cortisol levels in connection with birth of a newborn [8]. On the other hand, harmful levels of stress during labour and birth may be linked to adverse long-term changes including hyperactivity of the hypothalamic-pituitary-adrenal axis (HPA) [9]. There is also emerging evidence that supports for mothers during birth and a positive birth experience, as well as skin-to-skin contact between a mother and their newborn after birth, may be linked to sustained increased social interactive skills and reduced stress levels in the mother and infant. Oxytocin released during childbirth and skin-to-skin contact may play an important role in mediating these effects [4,5,10,11].

### 1.1. The Neuro-Endocrine Role of Oxytocin

Oxytocin, sometimes popularly referred to as the love hormone, is a vital and integrative component of a comprehensive neurochemical system that regulates positive interaction and exerts powerful anti-stress and restorative effects. Oxytocin is a small peptide consisting of nine amino acids. It is produced in the supraoptic and paraventricular nuclei (SON and PVN) of the hypothalamus. It is released into the circulation from the posterior pituitary to induce hormonal, peripheral effects. In parallel, oxytocin is released directly into the brain from the dendrites and axon collaterals (nervous extensions from the axons) of the oxytocin producing cells and also from oxytocinergic nerves originating from the PVN, where it regulates behavioural and physiological functions [12]. The oxytocinergic nervous system is very similar in both males and females. However, the female steroid hormones facilitate some functions of oxytocin giving rise to some slight differences between the sexes [13].

Oxytocin stimulates various kinds of social interactive behaviours including maternal behaviour and bonding [14,15]. It decreases fear and the sensation of pain and it may promote trust and well-being. In addition, oxytocin induces powerful anti-inflammatory and anti-stress effects including lowering of blood pressure, heart rate, and cortisol levels. In addition to decreasing the activity in the stress system (the hypothalamo-pituitary-adrenal axis and the sympathetic nervous system), it enhances the activity in the parasympathetic system [12,16].

The central and peripheral actions of endogenous oxytocin are executed by its receptor, which is a G protein coupled receptor. In humans, the oxytocin receptor gene is a single-copy gene consisting of four exons and three introns, localised at 3p25-3p26.2 [17]. The human oxytocin receptor gene (OXTR) has a specific DNA methylation-dependent regulatory region in its promoter, which is termed MT2. Increased methylation of MT2 is associated with reduced expression of OXTR. This suggests a regulatory role on gene transcription [18].

OXT exposure has been implicated in “hormonal imprinting”, which is the process by which exposure to a hormone in early life produces an enduring effect in the developing organism [19]. The perinatal period appears to be a sensitive period in terms of OXT’s actions within the brain, and this suggests that, following birth and across development, OXT can regulate a variety of social behaviours associated with bonding. Animal models have shown that OXT exposure at birth could influence subsequent social behaviour [19].

From a long-term perspective, oxytocin contributes to health by maintaining low levels of stress and inflammation, by stimulating restorative processes, and by inducing well-being and positive emotional engagement [4,20,21]. In addition, lack of oxytocin has been suggested to be linked with a lower quality of life [22]. The important role of oxytocin for mental health is supported by data showing that autism, social phobias, schizophrenia, and anxiety disorders are associated with decreased oxytocin functioning (lower release of oxytocin or lower oxytocin receptor function). In addition, some positive effects on these conditions have been observed in response to therapeutic administration of oxytocin as a nasal spray in clinical trials [23,24].

### 1.2. Oxytocin in Early Life, Animal Experiments

Experiments in sheep have shown that maternal oxytocin, released in connection with birth, is of importance for the development of maternal behaviour and bonding [15]. Administration of exogenous oxytocin also induces nurturing behaviours in labouring mothers, such as mice, voles, and other primates. It also induces long-term anti-stress and pain relieving effects [16]. Furthermore, animal experiments have shown that increased amounts of tactile stimulation, which released oxytocin during the first days after birth, stimulated social interactive behaviours, decreased fear, induced anti-stress effects, and stimulated growth [25,26]. The increased function of oxytocin receptors in the amygdala of the newborn may, in part, explain the decreased level of anxiety and the enhanced level of social interaction [27]. The stress reduction is partly mediated by a decrease in the activity of the HPA axis and of the sympathetic nervous system as evidenced by lower cortisol levels and a decrease in blood pressure [26,27,28]. These effects may be linked to long-term stimulation of the function of central alpha 2-adrenoreceptors [2,5]. Administration of oxytocin in adults and, as mentioned above, in newborn rats, has been associated with long-term anti-stress effects, including increased function in alpha 2-adrenoceptors in brain areas, which mediate inhibition of stress reactivity in response to oxytocin [13,20,26,29,30,31].

Taken together, these data support an important role of oxytocin in connection with the early postpartum period for promoting social interaction and for reducing anxiety and stress levels in the long term. Such effects of oxytocin may be exerted via oxytocin receptors or indirectly via other mechanisms activated by oxytocin. We are aware of epigenetic changes in animals. However, a mechanism which has been shown in animals might not necessarily be applicable to humans. We have not found any studies related to physiological birth in humans.

### 1.3. Oxytocin during Childbirth in Humans

Oxytocin plays a pivotal role in childbirth [11,32]. During labour, oxytocin is released in pulses into the maternal circulation, where it stimulates uterine contraction through its effect on oxytocin receptors on uterine smooth muscle [33]. During labour, the baby’s head promotes oxytocin release by exerting pressure on the cervix and vaginal wall, thereby activating the Fergusson reflex. Oxytocin is also released into the maternal brain during birth to decrease pain, fear, and stress and to promote social interaction. It may also contribute to the feelings of joy in connection with birth, by activation of the reward system [10,11].

During labour, oxytocin is also released in the baby; it is released into the circulation from the foetal posterior pituitary and into the brain from neurons within the brain. During labour, oxytocin in the foetus has been associated with pain relief and has been shown to counteract tissue damage caused by hypoxia due to its anti-inflammatory and restorative effects [11].

Oxytocin has also been demonstrated to be released into the circulation in response to skin-to-skin contact immediately after birth in both mothers and their infants [4]. The role of oxytocin released postpartum is, in addition to stimulating the expulsion of placenta, to further stimulate interaction and bonding between the mother and her newborn. In addition, prominent anti-stress, anti-inflammatory, and growth promoting effects are induced. In fact, skin-to-skin contact immediately after birth, with its potent anti-stress effects may be regarded as part of the normal birth process [4].

There is evidence that oxytocin released in connection with childbirth and during skin-to-skin contact after birth, also plays an important role in shaping long-term features of physiology and behaviour in human mothers and their newborns. These effects are discussed in more detail later in this article.

Given the long-term oxytocin-linked benefits regarding social interactive skills, stress levels, well-being, and health, one critical area for investigation is whether spontaneous vaginal birth followed by skin-to-skin contact could trigger epigenetic changes in the oxytocin system (the gene for oxytocin production or the gene for oxytocin receptors) in the mother and baby, which are, in turn, associated with optimal oxytocin function in the long term. The term epigenetics refers to changes in gene function that take place without a change in the DNA sequence. In most cases, these modifications turn genes on or off, permitting or stopping the gene from protein synthesis. Epigenetics encompass different mechanisms such as DNA methylation, histone modifications through acetylation, methylation or phosphorylation, and genomic imprinting [34].

This paper aimed at synthesizing existing research demonstrating epigenetic changes in the expression of the oxytocin and oxytocin receptor gene in association with physiological childbirth and immediate maternal/neonatal skin-to-skin experiences. A second aim of the study was to identify literature reporting long-term impacts on the mother and infant in association with childbirth and immediate maternal/neonatal skin-to-skin contact.

## 2. Methods

This was a scoping literature review where the primary question was whether epigenetic changes of oxytocin and its receptor mediate effects on the mother and infant during childbirth and skin-to-skin contact. We also searched the literature for evidence of other types of epigenetic changes in connection with childbirth. In addition, we searched for studies demonstrating the long-term effects of labour and skin-to-skin contact which were consistent with long-term effects of oxytocin.

The following databases: Pubmed, Medline, BioMed Central, Cochrane Library, OVID, and Web of Science, were searched using the following search terms: “oxytocin”, “oxytocin gene”, “oxytocin receptor”, “oxytocin receptor gene”, “oxytocin effect”, “vaginal birth”, “caesarean section”, “genetic”, “epigenetic”, “skin-to-skin contact”, without putting a limit on the date of publication. There were 896 articles identified which were written in English, which, following in-depth reading, only 69 were included, as the rest were excluded because they did not contribute to the focus of this review.

All articles were analyzed jointly by all the authors and the relevant data were extracted.

The findings from the retrieved literature are summarised below.

## 3. Results

### 3.1. Epigenetic Changes of Oxytocin or the Oxytocin Receptor Gene in Relation to Labour and Birth

In contrast to our hypothesis, we failed to identify any articles showing that epigenetic changes of the oxytocin gene or oxytocin receptor gene were induced in connection with birth or in response to skin-to-skin in humans.

We did, however, identify some studies that examined epigenetic mechanisms in relation to childbirth in humans. As mentioned above, epigenetics refer to changes related to gene function that take place without changing the existing DNA sequence. In most cases, these modifications turn genes on or off, permitting or blocking the gene from protein synthesis.

Epigenetic mechanisms have been demonstrated to be activated during birth. Four articles compared the level of global methylation in newborns after spontaneous birth and after caesarean section [35,36,37,38].

In one study conducted by Schlinzig et al. (2009), 37 newborn infants, of whom 21 were born spontaneously and 16 were delivered by elective CS, had blood sampled from the umbilical cord, three to five days after birth [35]. Leucocytes collected immediately after birth from infants born by caesarean section exhibited a significantly higher level of DNA methylation than did leucocytes of infants born by spontaneous vaginal birth (*p* < 0.001). The level of DNA methylation remained stable until three to five days later after vaginal birth, whereas it decreased in the caesarean section group (*p* = 0.01) and was no longer significantly different from that of vaginal birth.

Virani et al. (2012) carried out a large birth cohort study (*n* = 407) in which global DNA methylation in infants born by caesarean delivery was compared with the DNA methylation level of infants born by vaginal birth [36]. The level of methylation was measured in white blood cells from cord blood using two separate DNA methylation techniques (Luminometric methylation assay [LUMA] and LINE-1 methylation assay). At birth, there was no difference in global DNA methylation between caesarean delivery and vaginal delivery, nor was there any difference in global methylation between planned versus emergency caesarean section.

In a study by Almgren et al. (2014), the influence of the mode of birth on the epigenetic state in neonatal hematopoietic stem cells (CD34þ) was investigated [37]. In this study, 64 healthy, singleton, newborn infants born at term had cord blood sampled after elective caesarean or vaginal birth.

Similar to the results of Schlinzig et al. (2009) who studied methylation in neonatal leukocytes, CD34+ cells of babies born via caesarean section, the CD34+ cells were globally more DNA methylated as compared with babies born vaginally [35]. Maternal characteristics encompassing age, BMI prior to pregnancy, parity, length of labour, and duration of ruptured membranes, as well as infant characteristics including gestational age, birth weight, and sex were not found to be correlated with global DNA methylation in the neonatal stem cells.

A locus-specific analysis of single genes was also performed, which identified 343 loci with a significant difference in the level of DNA methylation of 10% or greater between vaginal birth and caesarean section. A majority (76%) of the differentially methylated loci in neonatal CD34þ cells was actually found to be hypermethylated after vaginal birth, which was in contrast with the findings related to global methylation where the level of methylation was more pronounced after caesarean section. In addition, the degree of DNA hypermethylation in three of the identified loci correlated to the duration of labour. The functional relevance of these differently methylated loci involved processes such as regulation of glycolysis, response to food, ketone metabolism, and immunoglobulin biosynthesis [37].

A prospective pilot study was carried out by Franz et al. (2014) on 41 newborn infants (23 with spontaneous birth and 18 delivered by elective caesarean section) [38]. Umbilical cord blood cells were collected after birth and investigated for global methylation. In addition, methylation of 96 single genes was measured. There was no difference in global methylation by mode of birth. However, the level of methylation of genes linked to T cell activation, cytokine production, inflammatory response, and stem cell transcription was significantly lower in newborn infants born vaginally, suggesting that these genes would be more active in infants born by vaginal birth as compared with those born by elective caesarean section [38].

To summarise, global hypermethylation was shown in one study after caesarean section, but not in the other studies, including the larger study performed by Virani et al. (2012) [36]. These four studies gave different results, and therefore it was difficult to judge the effect of vaginal labour versus CS on the amount of global DNA hypermethylation. In contrast, three specific loci were hypermethylated in the case of vaginal birth, and this effect correlated to the duration of labour [37]. These genes were specifically associated with the regulation of metabolic functions. Furthermore, the level of methylation of genes linked to T cell activation, cytokine production, inflammatory response, and stem cell transcription was significantly lower in newborn infants born vaginally, suggesting that these genes would be more active in infants born by vaginal birth as compared with those born by elective caesarean section [38]. These data might indicate that newborns born by vaginal birth have a better defense against inflammatory conditions [39].

Despite the lack of studies examining methylation of the oxytocin gene and its receptor in relation to labour, birth, and postnatal skin-to-skin contact, there is some suggestion from the included studies that the mode of birth could affect the epigenetic state of some other specific genes in neonatal blood cells, linked to metabolism, inflammation, and immune response. There were, however, no long-term studies that examined if these changes persisted over time, and, if so, what the clinical implications might be.

### 3.2. Polymorphisms of the Oxytocin Receptor

There are several polymorphisms of the oxytocin receptor in which different alleles may occur in some of the genes. Some of these minor structural differences in the oxytocin gene may be of clinical importance and have been shown to influence social interactive behaviours or stress reactivity. One example is a polymorphism in the rs53576 gene, where there can be either the G or A allele, which are linked to different psychological properties. Chen et al. (2011) showed that individuals with the G allele were able to deal more appropriately with stressors when given social support than those with the A allele [40].

In addition, some studies suggested that the effect on uterine contractions and the length of latent and active labour could be influenced by polymorphisms of the oxytocin receptor. In addition to parity and birth weight, polymorphism of the oxytocin receptor is one of the main predictors regarding length of active labour. An in vitro study on human myometrial cells showed that there was a significant difference between the amount of OXT stimulation used among reference and variant groups for rs4686302 (3.1 vs. 4.1 times, *p* = 0.022) and rs237888 (3.2 vs. 5.5 times, *p* = 0.001) [41].

A secondary analysis of birth records from 1229 pregnant women, who participated in a study on the effect of genetics on preterm labour and who had a vaginal birth later than 34-week gestation, was conducted [42]. Demographic, treatment data, and timed cervical examinations were collected from the participants’ electronic medical records. Three common OTR polymorphisms were genotyped. The relationship of all those factors on labour duration were investigated applying a mixed effect model. Women who expressed OTRrs2228485-T were found to have a significantly shorter latent labour, whereas women with OTRrs53576-A had a longer latent phase (*p* < 0.0001) as compared with the other polymorphisms. OTRrs2228485-T, OTRrs53576-A, ethnicity, and higher birthweight contributed independently to the longer latent phase [42].

A prospective study was performed on 187 women in order to investigate the occurrence of oxytocin gene receptor polymorphisms in women during childbirth requiring low (<4 mU/min) and high (>20 mU/min) oxytocin dosage. Thirty different OTR polymorphisms were identified, 10 of which were previously unknown and six of these were exclusive to women requiring high doses of oxytocin [43].

The results from these studies suggested that there could be a genetic factor to oxytocin sensitivity of the uterus in women giving birth. We decided to include these studies because they showed that small differences in the structure of the OXTR had an important effect regarding the function of oxytocin.This may in part explain why some women take longer to give birth physiologically, and perhaps even why some require induction or augmentation. More and larger studies are needed to examine thoroughly the association among specific OTR polymorphisms and the progress of labour. This could include an examination of any association among OTR gene variations and the length of active labour or whether pregnancy-related factors may contribute to the effect. It would also be of interest to investigate if these polymorphisms influence behavioural and physiological changes that occur during labour.

### 3.3. Impact of Caesarean Section on Oxytocin Levels

Although there were a few studies that examined physiological childbirth, and in spite of the negative findings regarding methylation of the oxytocin and oxytocin receptor gene in connection with birth, there was evidence of a disturbance of the oxytocin system in connection with caesarian deliveries, whether elective or emergency, as compared with spontaneous vaginal births.

Several studies have shown that oxytocin levels postpartum were significantly lower in both mothers and newborns after elective caesarean section as compared with vaginal birth [11,44]. A small-scale study in which repeated blood samples, allowing a detailed study of the release profile of hormones, were collected in connection with breastfeeding, reported that women who had a normal physiological birth had significantly more peaks of oxytocin and higher levels of prolactin in response to breastfeeding two days after birth than those giving birth by a caesarean section [45,46]. In addition the mothers who underwent caesarean section did not obtain the mental adaptations normally observed with the Karolinska Scale of Personality after vaginal birth, i.e., decreased levels of anxiety and increased levels of social interaction [46]. The low number of peaks was also associated with shorter periods of exclusive breastfeeding, suggesting that the number of peaks associated with breastfeeding had a predictive value for the duration of breastfeeding [10,45,46].

In addition, mothers who have given birth by elective caesarean section did not exhibit the normal release of oxytocin in response to skin-to-skin contact and breastfeeding immediately after birth, nor did they show the mental changes (recorded with the Karolinska Scale of Personality) of decreased levels of anxiety and increased social interaction two days after birth. Interestingly, these effects were restored if the mothers received intravenous oxytocin postpartum [47]. In fact, infusions of oxytocin postpartum reinforced oxytocin release in response to skin-to-skin contact in women who had an elective caesarean section, and they also reinstated the mental adaptive changes normally induced by birth.

The results of these studies support the important role of oxytocin release in connection with birth and postpartum, with consequences for the psychological adaptations and also for the release of prolactin, and thereby for breastfeeding. In addition, these studies point to a malfunction of the oxytocin system in mothers who have given birth by caesarean section. These results may explain why caesarean section, in particular elective sections, could be linked to breastfeeding problems, as has been demonstrated in several studies [44,45,48,49,50,51]. These breastfeeding problems could, at least in part, be due to the lower activity in the oxytocin system in the women having given birth by caesarean section.

### 3.4. Longterm Impact of Mother and Baby Interaction Immediately after Birth

Klaus et al. (1972) showed that early contact between a mother and their newborn promoted social interaction between the mother and infant for several months after birth and based on these observations the term “the early sensitive period” was coined, in this way describing a short period after birth during which closeness between mother and newborn influences the behaviour of the mothers and their newborn in a positive direction in the long term [52].

On a similar note, a systematic review on early skin-to-skin contact for mothers and their healthy newborn infants, revealed that babies who were put skin-to-skin made more physical contact with their mothers and cried less, indicating decreased levels of pain and fear [53,54]. Skin-to-skin contact after birth had a positive effect on maternal attachment, babies breastfed for a longer period of time and cried less, and late preterm infants were noted to have better cardiopulmonary stability [55].

Bystrova and colleagues (2009) studied whether different labour ward routines, in particular, skin-to-skin contact and separation in the labour ward, as well as, during the maternity stay, rooming-in versus nursery care in the maternity ward, affected the mother–infant interaction one year postpartum. The effects of these interventions on mother-infant interaction one year postpartum were studied using the Parent Child Early Relational Assessment (PCERA). Findings from this study revealed that contact between the mother and the infant in the initial two hours following birth appeared to be noteworthy for maternal sensitivity, for mutuality in the dyad, and for infant’s self-regulation or ability to handle stress at the time when the infant was one year old as measured by PCERA. These effects seemed to be consequential to the skin-to-skin contact between mother and infant in the initial two hours (early sensitive period) [56]. However, early suckling, an alternative way of sensory stimulation during the two first hours after birth seemed to make up for the absence of skin-to-skin contact. The study concluded that sensory contact between mother and newborn in the early sensitive period resulted in long-term positive effects on mother–infant interaction and decreased the infants stress reactivity [56].

**Figure 1:**
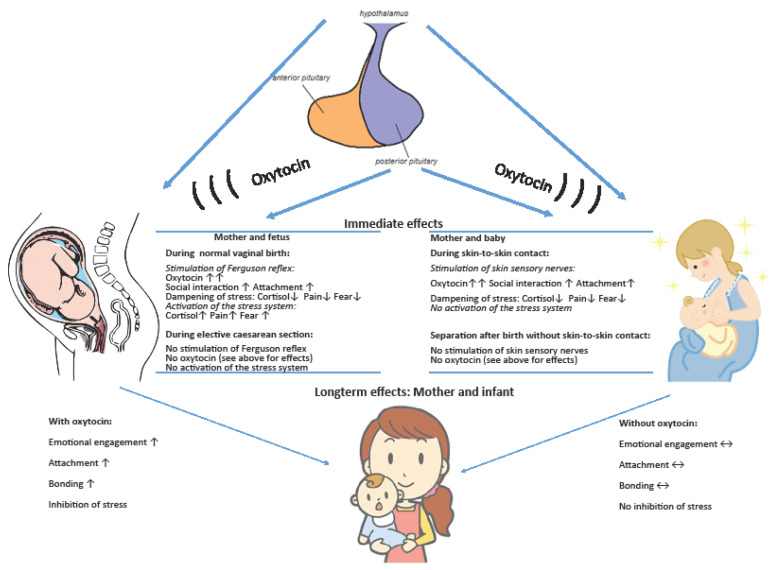
The immediate and longterm effects of oxytocin on the mother and infant during childbirth and skin-to-skin contact.

Oxytocin is released during skin-to-skin contact after birth in both mother and baby [11] (Figure 1). This release, to a large extent, is mediated by activation of cutaneous sensory nerves in response to warmth, light pressure, and stroking of the skin [4]. As skin-to-skin contact is associated with oxytocin release in both mother and infant, the experimental effects cited above suggest that physiological oxytocin release during skin-to-skin contact in both the mother and newborn, might contribute to some of the long-term effects noted up to one year of age. The long-term effects caused by oxytocin, may potentially be induced by a sustained increase in oxytocin release or via enhanced function of the oxytocin receptor.

## 4. Discussion

In this literature review, no studies that demonstrated epigenetic changes of oxytocin and its receptor in connection with human childbirth, were identified. Data regarding global methylation of genes in connection with childbirth, were identified. In addition, some polymorphisms of the oxytocin receptor were identified.

The hypothesis, put forward in this review, was that the function of the oxytocin system is influenced in connection with childbirth, in order to induce long-term beneficial adaptations for both mother and neonate (Figure 1). Labour, birth, and the practice of skin-to-skin contact after birth are linked to large amounts of oxytocin release, and to both immediate and long-term behavioural and physiological effects. During birth, pain is relieved and the levels of fear and stress are reduced by oxytocin and these effects are further enhanced by skin-to-skin contact [10,11]. Some of these effects have been demonstrated to be transformed into long-term effects, as discussed previously [52].

Oxytocin released in response to the Fergusson reflex in connection with birth and in response to activation of sensory nerves in the skin during skin-to- skin contact in human mothers and their foetuses/infants is likely to contribute to these short and long-term psycho-physiological adaptations [11]. Epigenetic changes of the oxytocin gene or the oxytocin receptor gene in connection with birth might potentially explain how the short-term effects are transferred into long-term effects. However, we did not identify any studies showing epigenetic changes in oxytocin and oxytocin receptor genes. In contrast, increased levels of oxytocin receptor DNA methylation have been shown to occur during pregnancy, and these levels have been associated with an increased risk of postpartum depression in women [57].

We did identify some studies that showed differences in the level of global methylation of genes as compared with caesarean section and vaginal birth, but these results were conflicting [35,36,38]. Some studies in which the level of methylation of single genes was measured showed that some genes reflecting metabolism, inflammation, etc. were more or less methylated, depending on the type of birth or duration of vaginal birth.

One reason for not picking up any epigenetic changes in the oxytocin gene or the oxytocin receptor gene might be that oxytocin often exerts its effects in the following two steps: first by activation of the oxytocin receptor, which in turn interacts with other signalling systems, such as the glucocorticoid (GC) receptor [16]. Therefore, the intense stimulation of oxytocin release and of its receptor during birth and skin-to-skin contact may induce sustained long-term effects via influencing the function of such secondary mechanisms. Therefore, another option is to search for epigenetic changes “downstream” to the oxytocin receptor, i.e., in the systems/functions influenced by the high levels of oxytocin during birth. Oxytocin has been shown to promote growth and reduce stress activity in the long term by stimulating central alpha-2 adrenoceptors, which exert potent inhibitory effects on the central noradrenergic neurons emanating from the locus coeruleus (LC) [58]. In this way, increased oxytocin activity may exert anti-stress effects and decrease the activity of the HPA axis and of the sympathetic nervous system [12]. Various types of stressors have been shown to induce epigenetic changes in the GC receptor [59]. Oxytocin can modulate the function of the GC receptor [29,30]. The concept of indirect effects via other signaling systems is supported by evidence demonstrating downregulation of the activity of the HPA axis, and of the function of the GC receptor, due to prior activation of oxytocin release and of oxytocin receptors [59].

Oxytocin exerts effects on a mother and newborn during and following childbirth. Many of these effects are absent, or are less evident, in a mother and her neonate having had an elective caesarean section. The practice of skin-to-skin contact after birth increases the expression of the oxytocin-linked effects. In fact, skin-to-skin contact immediately after birth could be regarded as a part of birth, when stress levels, which are high during birth, are decreased. To date, it is still not clear by which mechanism oxytocin might beneficially induce its long-term effects. We speculate that the oxytocin system plays a major role, but the effect could be indirect as oxytocin is involved in the regulation of many other transmitter systems, including the HPA axis [4]. This is further supported by the fact that epigenetic changes of the stress axis and the GC receptor do occur as a response to stressful situations and experiences [60]. Oxytocin may act to buffer and antagonise such effects.

Given the positive effects of oxytocin, it is important to allow the oxytocin-induced effect in connection with physiological birth to be expressed. This is because oxytocin will stimulate social skills and decrease stress levels and inflammation, and therefore is linked to positive physical and mental health in the long term. These points of reflection are of importance when choosing the mode of birth and implementing possibly unnecessary interventions during birth, as many medical interventions are linked to a decreased amount of oxytocin release in connection with childbirth.

## 5. Conclusions

This review was designed to locate and describe current evidence regarding epigenetic changes of the oxytocin gene and the oxytocin receptor gene in connection with childbirth in humans, and the potential consequences of any such changes for mother and baby in the short and long term. We did not find any specific studies regarding epigenetic changes of the oxytocin and the receptor gene in connection with childbirth or skin-to-skin contact. We did find some evidence that methylation might result from events during labour and birth, both globally and for specific genes, though this was not consistent among studies. Given that high amounts of oxytocin are released in connection with birth, there is a large body of evidence that supports the important role of oxytocin on effective labour; and there is strong evidence for oxytocin’s impact on pro-social behaviour and anti-stress effects in both mothers and newborns. This is a critical area for further investigation.
